# Limitations of human brain organoids to study neurodegenerative diseases: a manual to survive

**DOI:** 10.3389/fncel.2024.1419526

**Published:** 2024-07-09

**Authors:** Nerea Urrestizala-Arenaza, Sonia Cerchio, Fabio Cavaliere, Chiara Magliaro

**Affiliations:** ^1^Achucarro Basque Center for Neuroscience, The Basque Biomodels Platform for Human Research (BBioH), Leioa, Spain; ^2^Centro di Ricerca “E. Piaggio” – University of Pisa, Pisa, Italy; ^3^Fundación Biofisica Bizkaia, Leioa, Spain; ^4^Department of Information Engineering, University of Pisa, Pisa, Italy

**Keywords:** brain organoids, neurodegenerative diseases, Alzheimer’s disease, Parkinson’s disease, amyotrophic lateral disease

## Abstract

In 2013, M. Lancaster described the first protocol to obtain human brain organoids. These organoids, usually generated from human-induced pluripotent stem cells, can mimic the three-dimensional structure of the human brain. While they recapitulate the salient developmental stages of the human brain, their use to investigate the onset and mechanisms of neurodegenerative diseases still faces crucial limitations. In this review, we aim to highlight these limitations, which hinder brain organoids from becoming reliable models to study neurodegenerative diseases such as Alzheimer’s disease (AD), Parkinson’s disease (PD), and amyotrophic lateral sclerosis (ALS). Specifically, we will describe structural and biological impediments, including the lack of an aging footprint, angiogenesis, myelination, and the inclusion of functional and immunocompetent microglia—all important factors in the onset of neurodegeneration in AD, PD, and ALS. Additionally, we will discuss technical limitations for monitoring the microanatomy and electrophysiology of these organoids. In parallel, we will propose solutions to overcome the current limitations, thereby making human brain organoids a more reliable tool to model neurodegeneration.

## 1 Introduction

Studying neurodegeneration is a global challenge in our aging society. For instance, diagnoses of Alzheimer’s and Parkinson’s diseases are predicted to double in the next 30 years (Dorsey et al., 2018). Identifying early-stage biomarkers for prompt diagnosis and treatment will have a tremendous impact on our economy and society. Traditionally, the study of human neurodegeneration has relied on post-mortem brain samples or longitudinal clinical studies. However, these methodologies have significant limitations: post-mortem samples only allow for the study of the final stage of the disease, while longitudinal studies require large periods, which may not be compatible with the urgent challenges in this field of research.

**Figure 1:**
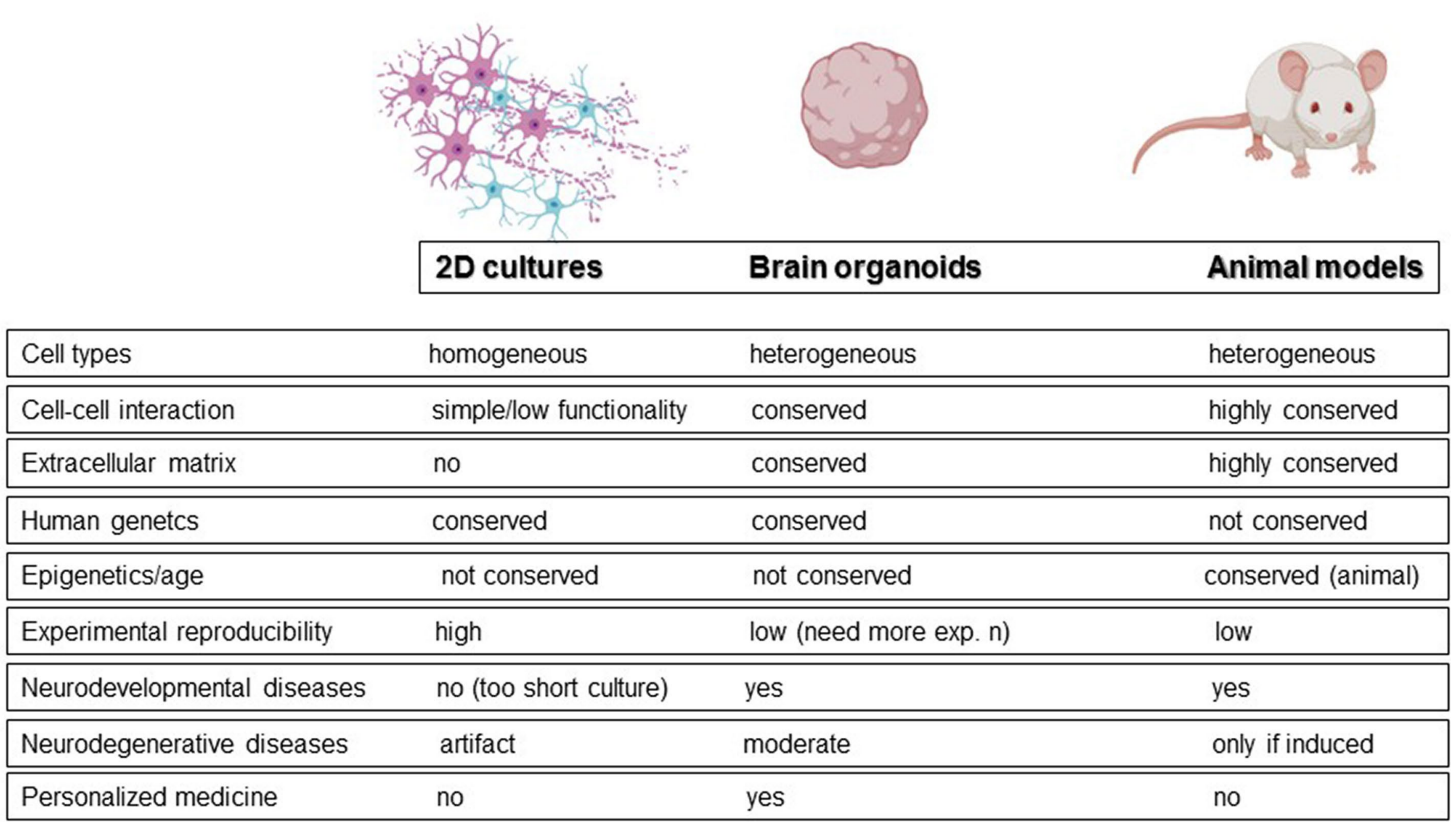
Cell and animal models used to study neurodegenerative diseases with main features.

Animal models and monolayer cell cultures represent the alternatives to post-mortem and longitudinal studies. Animal models, although useful for understanding some molecular mechanisms, do not accurately reflect human brain physiology. To date, wild-type animals can mimic only some of the salient features observed in neurodegeneration, like for example reactive gliosis. For example, no senile plaques have been observed in aged mice (Hall and Roberson, 2012). As a result, neurodegeneration is usually induced in animals through toxins. On the other hand, traditional monolayers can be generated with human cells but have inherent limitations in physiological relevance, such as unrealistically flattened dendrites (Fabbri et al., 2023). In this context, brain organoids have been proposed as a promising alternative for studying neurodegeneration and bridging the gap between patient research and model organisms (Figure 1; Adlakha, 2023; Jusop et al., 2023). These 3D cultures allow cells to establish more physiological connections, promoting cell division, extracellular matrix synthesis, and the acquisition of morphology and gene expression patterns that more closely resemble those of a human brain (Tekin et al., 2018). Moreover, because generated from pluripotent stem cells, they maintain the genetic fingerprint of the donor cells, potentially enabling the exploration of neurodegeneration from a patient-oriented perspective. Neural organoids can be classified into two broad categories: unguided neural organoids and regionalized neural organoids, obtained by activation of specific guidance signaling during differentiation. In unguided organoids, the absence of guidance results in significant cell diversity, including cells derived from neural lineage and non-neural derivatives, leading to higher variability and more complex reproducibility. In contrast, regionalized neural organoids are designed to mimic specific domains of the nervous system at the anatomical, cellular, or molecular level. For example, “midbrain organoids” are regionalized organoids that mimic the midbrain.

The range of their applications is broad, including neurobiological basic research, drug discovery, gene therapy, cell therapy, precision and regenerative medicine (Park et al., 2021; Zhao et al., 2022; Kim and Chang, 2023; Giorgi et al., 2024).

However, brain organoids primarily serve as neurodevelopmental models that recapitulate early human brain development. Several groups have demonstrated neuronal senescence in brain organoids, whether genetically induced or resulting from long-term culture (Aguado et al., 2021; Shafqat et al., 2023). However, the difficulty or practical limitation in generating a functional microcirculation, the extension of a necrotic core over time in culture, and the massive presence of embryonic markers in mature organoids (e.g., Sox2) suggest that organoids cannot activate a specific aging program and therefore cannot be used as a reliable model for human brain aging (Andrews and Kriegstein, 2022). Additionally, since pluripotent stem cell reprogramming maintains the genetic but not the epigenetic fingerprint, brain organoids can be generated from donor patients with familial neurodegenerative diseases (NDD), while sporadic forms can only be induced.

To improve the model structurally and functionally, recent efforts have focused on “assembling” brain organoids with various cell types or entire organoids. Functional cortico-motor assembloids were first produced and characterized in 2020, opening new possibilities for better modeling NDD such as amyotrophic lateral sclerosis (Andersen et al., 2020). However, creating more complex structures significantly impacts the reproducibility of the constructs, as coordinating proliferation and differentiation of different cell types in multicellular or multi-tissue organoids is challenging (Zhao et al., 2022).

**Figure 2:**
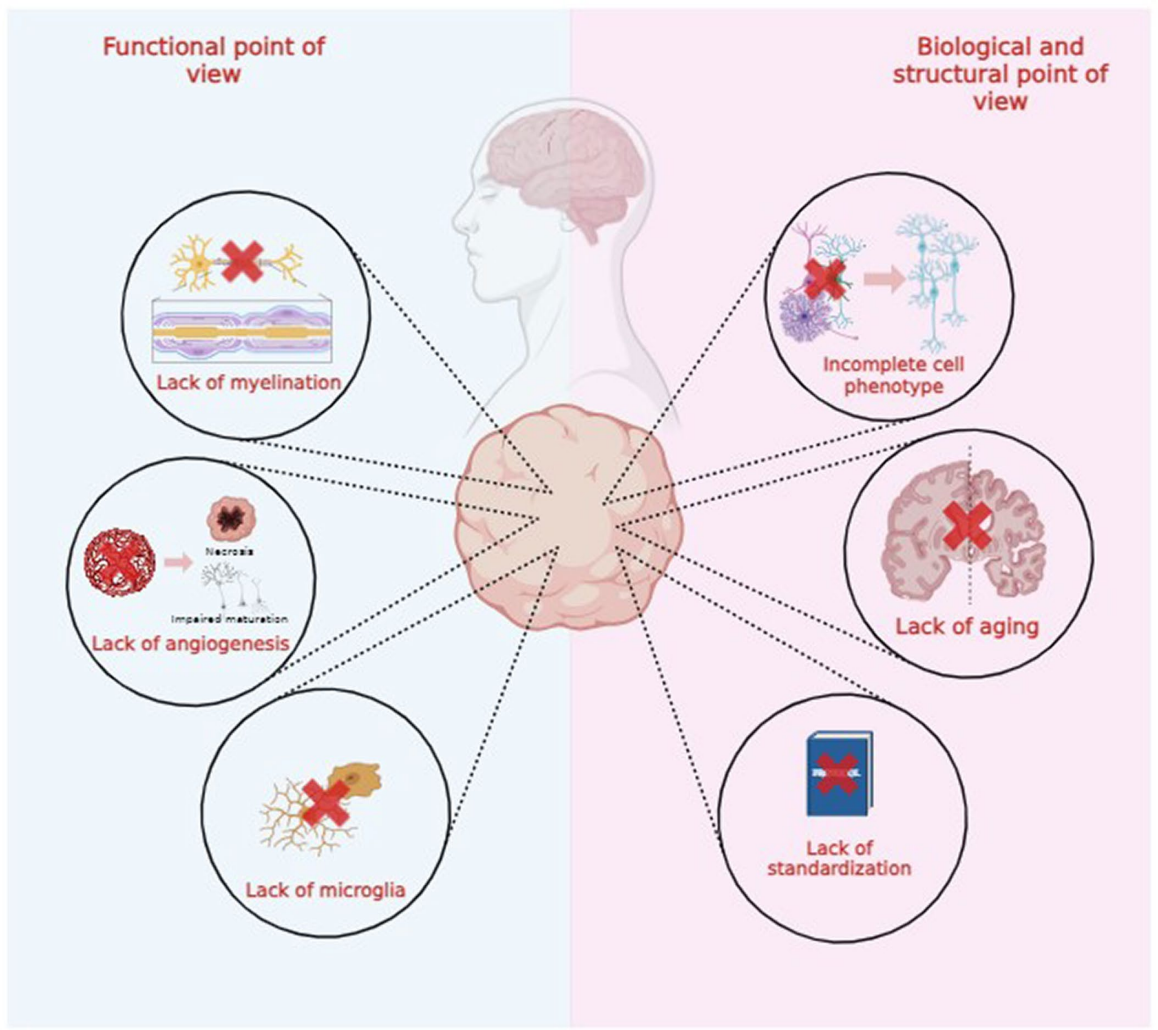
Functional, biological and structural limitations of brain organoids technology.

Both brain organoids and assembloids cannot be considered exact replicas of healthy or degenerated brains due to several functional, structural, and biological limitations, as illustrated in Figure 2. In this review, we explore these limitations to advance technological and scientific progress in the field (Poli et al., 2019; Eichmüller and Knoblich, 2022; Kim and Chang, 2023).

## 2 Brief overview on functional assays

Brain organoids are faithful representations of human brains, mimicking their cellular and molecular structure. However, replicating the complex neuronal circuitry precisely remains challenging. While transcriptomic analysis has led to significant advances in characterizing the cellular complexity of brain organoids (Quadrato et al., 2017), improved functional and electrophysiological analyses are still needed to define the full potential of brain organoids. As already discussed by Poli and colleagues, different experimental approaches have been adopted for functionally charactering brain organoids (Poli et al., 2019). Among all, high-density multi-electrode arrays (HD-MEA) have been used to better characterize the functional activity of different organoid regions. High-density analysis, with more than 24,000 electrodes, allows for the precise electrical mapping of neurons at depths up to 100 μm (Sharf et al., 2022). This functional analysis can be used to characterize the spontaneous activity as well as pathological and pharmacological alteration of the neuronal activity. Samarasinghe and colleagues demonstrated highly abnormal and epileptiform-like activity in organoids derived from Rett syndrome iPSCs, accompanied by transcriptomic differences (Samarasinghe et al., 2021). It is worth highlighting that even with an increased number of electrodes, HD-MEAs are planar and can only record a small fraction of three-dimensional organoids. An evolution of the MEA has been proposed recently using a 3D basket-like electrode configuration known as Kirigami electronics (Yang et al., 2024). Kirigami electronics is a basket with electrodes that allows the chronic recording of organoids in suspension for up to 120 days. This system preserves the organoids’ morphology, cytoarchitecture, and cell composition during cultures, providing several advantages, such as the detection of disease-associated electrophysiological phenotypes and network connectivity.

## 3 Current models for neurodegenerative diseases

NDD are generally characterized by chronic neuronal loss of unknown etiology and multifactorial origins. The principal risk factor is age (e.g., for Alzheimer’s disease, Parkinson’s disease, or frontotemporal dementia); however, other factors such as pollution, genetics, and lifestyle can trigger neurodegeneration many years before clinical symptoms appear, making it difficult to identify tools for early diagnosis (Livingston et al., 2020; Cavaliere and Gülöksüz, 2022). Moreover, there are no cures for most NDDs. The most common NDDs are Alzheimer’s disease (AD), Parkinson’s disease (PD), and amyotrophic lateral sclerosis (ALS). Below, we present several strategies to model NDD in brain organoids, though these models are still far from accurately replicating the pathology, particularly due to the lack of a specific aging program and the multifactorial astrocytic reaction to neuropathology.

### 3.1 Alzheimer’s disease

Alzheimer’s Disease (AD) is characterized by a progressive loss of memory and cognitive functions. Only 5–10% of cases are considered familial (FAD), involving different deterministic genes such as presenilin (PSEN) and amyloid precursor protein (APP), or risk genes like apolipoprotein E (APOE). However, age is the major risk factor in sporadic AD (SAD), with evident sexual dimorphism (almost 70% of patients older than 60 years are women) (Cerneckis et al., 2023). The principal neuropathological hallmarks of AD are the presence of Amyloid-β (Aβ) plaques and tau-positive neurofibrillary tangles, though the role of these structures in the onset of AD is still debated. The damage seems to start in the entorhinal cortex and the hippocampus, regions crucial for memory formation. Additionally, neuroinflammation appears to be involved in the onset and propagation of AD (Leng and Edison, 2021).

Brain organoids generated from patients with genetic variants recapitulate some molecular hallmarks, such as Aβ aggregation or tau hyperphosphorylation (Bubnys and Tsai, 2022). Several studies have demonstrated that mutated tau induces impaired proteostasis, neuroinflammation, cholesterol deregulation, and selective loss of glutamatergic neurons in brain organoids (Bowles et al., 2021; Glasauer et al., 2022). Genetic mutations can also be induced exogenously through gene editing of hiPSCs. AAV-mediated overexpression of Aβ or tau (Cerneckis et al., 2023), as well as the isogenic generation of APOE3 and APOE4, provides important information about the molecular mechanisms triggering neuronal death (Huang et al., 2022). Overexpression of the mutant tau-P301L in brain organoids can lead to the formation of oligomers and fibrils, although hiPSCs lack the same tau isoform expressed *in vivo*, leading to different aggregate shapes.

Some attempts at modeling sporadic AD have been reported in the literature. Park and colleagues observed the same levels of soluble Aβ40, Aβ42, and phospho-tau in brain organoids as those observed in patients with sporadic AD using positron emission tomography (Park et al., 2021). Moreover, overexpressing APOE4 isoform by CRISPR-Cas9 (Lin et al., 2018; Zhao et al., 2020) mimics salient features of sporadic AD, including increased Aβ production, reduced microglial phagocytosis, and increased inflammatory response. An interesting model has been proposed by treating brain organoids with AFTIN-5 (Pavoni et al., 2018), an activator of Aβ42, which induces the accumulation of both Aβ oligomers and their target, the cellular prion protein, thus resembling the chronic accumulation of Aβ described in *in vivo*. However, the loss of epigenetic fingerprint following hiPSC reprogramming makes modelling sporadic AD more difficult to achieve. Some attempts at exploiting assembloids, in particular cortical-blood vessel ones, to provide vasculature to brain organoids, showed an increased expression of microglia and astrocytes, and β-amyloid plaques (Kong et al., 2023).

### 3.2 Parkinson’s disease

Parkinson’s Disease (PD) is a chronic and multifactorial NDD characterized by a broad spectrum of motor symptoms, such as tremor, stiffness, walking difficulties, lack of balance, and coordination problems. It involves the progressive loss of dopaminergic neurons in the substantia nigra pars compacta and further degeneration of the striatum. The main neuropathological hallmark of PD is the presence of neuronal inclusion bodies—Lewy bodies—whose main component is the misfolded α-synuclein. A key question in PD neurodegeneration is what causes the selective death of these neurons.

Midbrain organoids are the most widely used models to study PD PD (Jo et al., 2016; Zagare et al., 2022). By using a specific small molecule cocktail, Tyrosine hydroxylase positive neurons (TH+) can be generated by activating midbrain progenitor cells with BDNF, GDNF, and FGF8 (Smits et al., 2019). Genetic PD can be modeled in midbrain organoids using iPSCs derived from patients. It has been demonstrated that midbrain organoids generated from LRRK2(G2019S) or PRKN patients (Kim et al., 2019a) recapitulate the principal hallmarks of neurodegeneration observed *in vivo* and show alterations in novel biochemical pathways with abnormal phenotypes of LRRK2 sporadic PD and alpha-synuclein-mediated gene alteration. Midbrain organoids generated from patient iPSCs show a significant decrease in TH+ neurons and, consequently, a loss of dopamine release. These organoids also exhibit impaired α-synuclein translocation from the cytoplasm (in immature neurons) to the synaptic terminals (in mature neurons), suggesting an alteration in the functional role of α-synuclein and altered synaptic transmission.

Advanced gene editing technologies, such as CRISPR-Cas9, have also been utilized. Recently, Kim et al. (2023) developed an optogenetics-assisted α-synuclein aggregation induction system, which can rapidly induce α-synuclein aggregation and neuronal death. Although instrumental in studying the molecular mechanisms underlying dopaminergic alterations, midbrain organoids cannot model the cortical-nigro-striatal pathway involved in PD progression. To address this, assembloid models have been designed. Notably, cortico-striatal (Miura et al., 2020) and brain-striatal (Barmpa et al., 2023) assembloids showed axonal projections originating from and directed toward the striatum, catecholamine release from the midbrain to striatum and functional synapse formation- features crucial for better modelling PD.

In a more recent study, the use of 3xSNCA midbrain organoids helped to shed light into the role of astroglia in the development of PD neurodegeneration. The authors demonstrated the association between the accumulation of pathogenic α-synuclein in midbrain organoids and the acquisition of a senescent phenotype in astrocytes, supporting the hypothesis of the loss of homeostatic support proposed by Ramos-Gonzalez et al. (2020) and Muwanigwa et al. (2024).

Recent works integrate hiPSC-derived microglia to study neuroinflammation, which is known to be implied in PD onset and progression (Sabate-Soler et al., 2022). These studies showed that the integration of microglia is associated with reduced cell death in the necrotic core, reduced oxidative-stress-related gene expression, synapse remodeling, and functional maturation of neurons.

### 3.3 Amyotrophic lateral sclerosis

Amyotrophic Lateral Sclerosis (ALS) is a progressive NDD of unknown etiology, affecting the neuromuscular system and rapidly leading to respiratory failure and death, usually within 3–5 years from the onset of symptoms. The neuropathological hallmark in sporadic cases is the accumulation of TDP-43 protein in motor and non-motor neurons (Jo et al., 2020). Both organoids and neuro-muscular assembloids have been proposed to study the molecular and cellular mechanisms of ALS.

Sliced brain organoids generated from patient cells harboring the C9ORF72 hexanucleotide repeat expansion mutation and cultured in an air-liquid interface for more than a year showed disturbances in transcription, proteostasis, and DNA repair in both astrocytes and neurons (Szebényi et al., 2021). Slicing the organoids presents the advantage of reducing the necrotic core by exposing it to nutrients and oxygen. This long-term organoid model allowed the characterization of excessive astrocytic autophagy and the accumulation of neuronal dipeptide repeat protein poly(GA), leading to apoptosis. ALS-derived organoids were used to demonstrate a novel set of misregulated RNA targets in TDP-43-overexpressing neurons and in patients with TDP-43 proteinopathies (Hruska-Plochan et al., 2024). Neurons within the organoids showed a loss of nuclear TDP-43 and the upregulation of the synaptic protein NPTX2, linking the two proteins to neurotoxicity in ALS.

To better model the neuromuscular degeneration observed in ALS patients, ecto-mesodermal progenitor cells (EMPC) have been used to generate neuromuscular (or sensorimotor) assembloids. hPSC-derived axial stem cells, the EMPCs of the posterior body, generate both spinal cord neurons and skeletal muscle cells that self-organize to form human neuromuscular organoids. Neuromuscular organoids show neuromuscular junctions, Schwann cells, a microglia-like population, and recapitulate muscular asthenia in patient-derived or isogenic assembloids (Faustino Martins et al., 2020; Pereira et al., 2021; Hong et al., 2023). Additionally, organoids have been successfully employed to study possible therapeutic strategies, particularly to test GSK2606414, a potent PERK inhibitor previously demonstrated to be effective against TDP-43-associated neurodegeneration (Kim et al., 2014).

## 4 Structural, functional, and biological limitations

### 4.1 Structural and biological limitations

#### 4.1.1 Lack of standardization

Reproducibility is one of the biggest challenges in cerebral organoid and assembloid technology today. Variability can stem from both experimental procedures and biological materials. Typically, these procedures include several steps, many of which are poorly described, highly operator-dependent, and require skilled users and manual expertise. Simplifying the dorsal forebrain organoid protocol has been shown to increase reproducibility (Velasco et al., 2019). For example, Ha et al. successfully produced homogeneous midbrain-like constructs capable of reducing experimental variability by simplifying the midbrain organoids generation protocol for modeling PD (Ha et al., 2020).

Biologically, the composition of commercially available media, batches of serum and extracellular matrix, levels of growth factor purity, and the presence of mycoplasma contamination can all result in poor reproducibility. Brain organoids are often encapsulated in biologically derived matrices that mimic the extracellular environment and contribute to mechano-sensing signaling, such as Matrigel, which is quite heterogeneous among lots (Aisenbrey and Murphy, 2020). To reduce the variability from Matrigel and other commercial matrices, new synthetic hydrogels have been proposed as an alternative and standardizable support. For instance, the poly(lactic-co-glycolic acid) copolymer (PLGA) fiber microfilament-based floating scaffold has been implemented for the growth of embryoid bodies and is associated with increased reproducibility (Lancaster et al., 2017). Similarly, soluble factors such as growth factors can exhibit batch-to-batch variability (Zhao et al., 2022).

Reproducibility is also influenced by several culture parameters, including the source of initiating cells (e.g., iPSCs, neural precursor cells, or embryonic cell lines), the isolation and purification methods, and the protease used in the dissociation procedure (Zhao et al., 2022). Genetics and epigenetics may pose a significant risk of variability for hiPSCs, as the reprogramming method, whether viral or episomal, can yield different outcomes in different clones, resulting in diverse phenotypes (Allison and Lowry, 2017). This variability is even more pronounced when the cells carry mutations associated with diseases of variable severity and penetrance, such as PD, AD, ALS, and FTLD (Van Deerlin, 2012). Furthermore, stem cell differentiation and self-assembly capacity are naturally stochastic, increasing the rate of heterogeneity. This is more evident in non-guided organoids since “guided” protocols strictly control cellular specialization. In this regard, stem cells can be engineered to express specific surface proteins, peptides, or polymers to achieve finer control over cellular assembly (Zhang and Kohn, 2012). Gene editing may also be used to reprogram cellular responses to external stimuli and determine their fate, although genetically modified organoids might decrease their physiological relevance (Hofer and Lutolf, 2021).

#### 4.1.2 Incomplete cell phenotyping

In 2020, Bhaduri et al. performed an advanced high-throughput single-cell RNA sequencing, comparing the transcriptome of primary human cortical cells from various developmental periods and brain areas, with those of cortical organoid cells (Bhaduri et al., 2020). The comparative analysis showed that both samples expressed markers from a wide range of cell types, including radial glia, intermediate progenitors, maturing neurons, and interneurons. However, the transcriptomic analysis revealed reduced subtype resolution with a smaller number of high-quality cellular subtypes in organoids. This lower resolution was attributed to impaired cellular maturation, as maturation marker genes such as MEF2C and SATB2 are upregulated only in primary cells.

It is worth noting that the timing of neurogenesis in a dish differs from that observed *in vivo*. For example, the fibrous structure supporting neuronal migration during neurogenesis is observed after 15 weeks of development in the primary human cortex compared to 5 weeks in organoids. Thus, while neural progenitor specification occurs in a slower and broader manner *in vivo*, generating well-defined cellular identities, maturation is not properly activated in organoids: it is fast, inaccurate, and not homogeneous among cells. These *in vivo* vs. *in vitro* discrepancies might be attributed to cellular stress, particularly ER and metabolic stress, as evidenced by the upregulation of genes such as PGK1, ARCN1, and GORASP2 (Bhaduri et al., 2020). Indeed, ER stress has been previously linked to inhibition of cell-type specification (Laguesse et al., 2015). Transplantation of 8-weeks old brain organoids into postnatal day 4 mice rescues the expression of cellular stress markers in the constructs, suggesting that maturation is a consequence of environmental conditions, such as the absence of non-neuronal cell types and vasculature.

**Figure 3:**
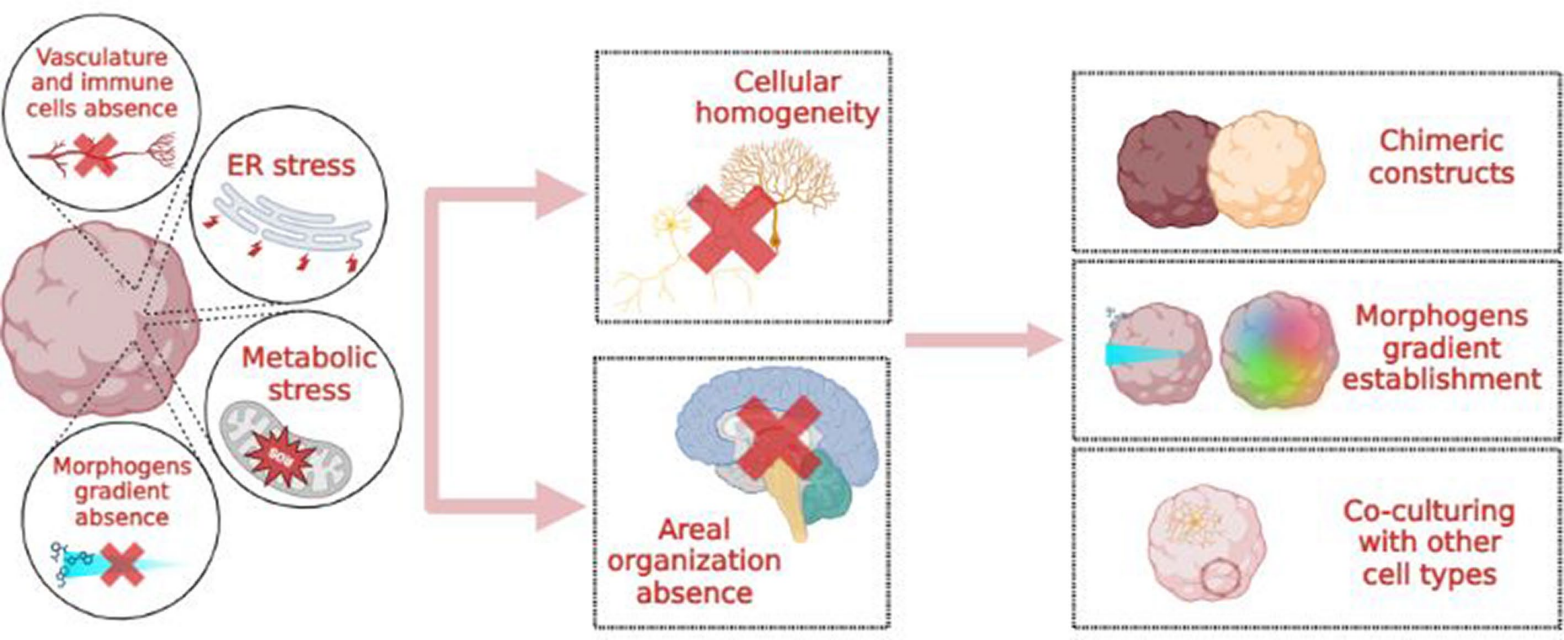
Causes, consequences and possible solutions to the “cellular homogeneity” issue in brain organoids.

Besides the integration and co-cultivation of brain organoids with other cell types, such as endothelial and immune cells, a possible solution would be to more closely mimic *in vivo* conditions. *In vivo*, excitatory and inhibitory cortical neurons originate from different germinal zones: the dorsal and ventral telencephalon, respectively. Consequently, a protocol encompassing only “dorsalizing” signals will produce “all excitatory” organoids with a reduced percentage of inhibitory interneurons. Conversely, achieving cellular and topographical complexity may involve creating morphogen gradients or three-dimensional chimeric constructs, such as the fusion of dorsal and ventral forebrain spheroids (Figure 3; Birey et al., 2017; Sloan et al., 2018).

#### 4.1.3 Lack of adult maturation and aging process

**Figure 4:**
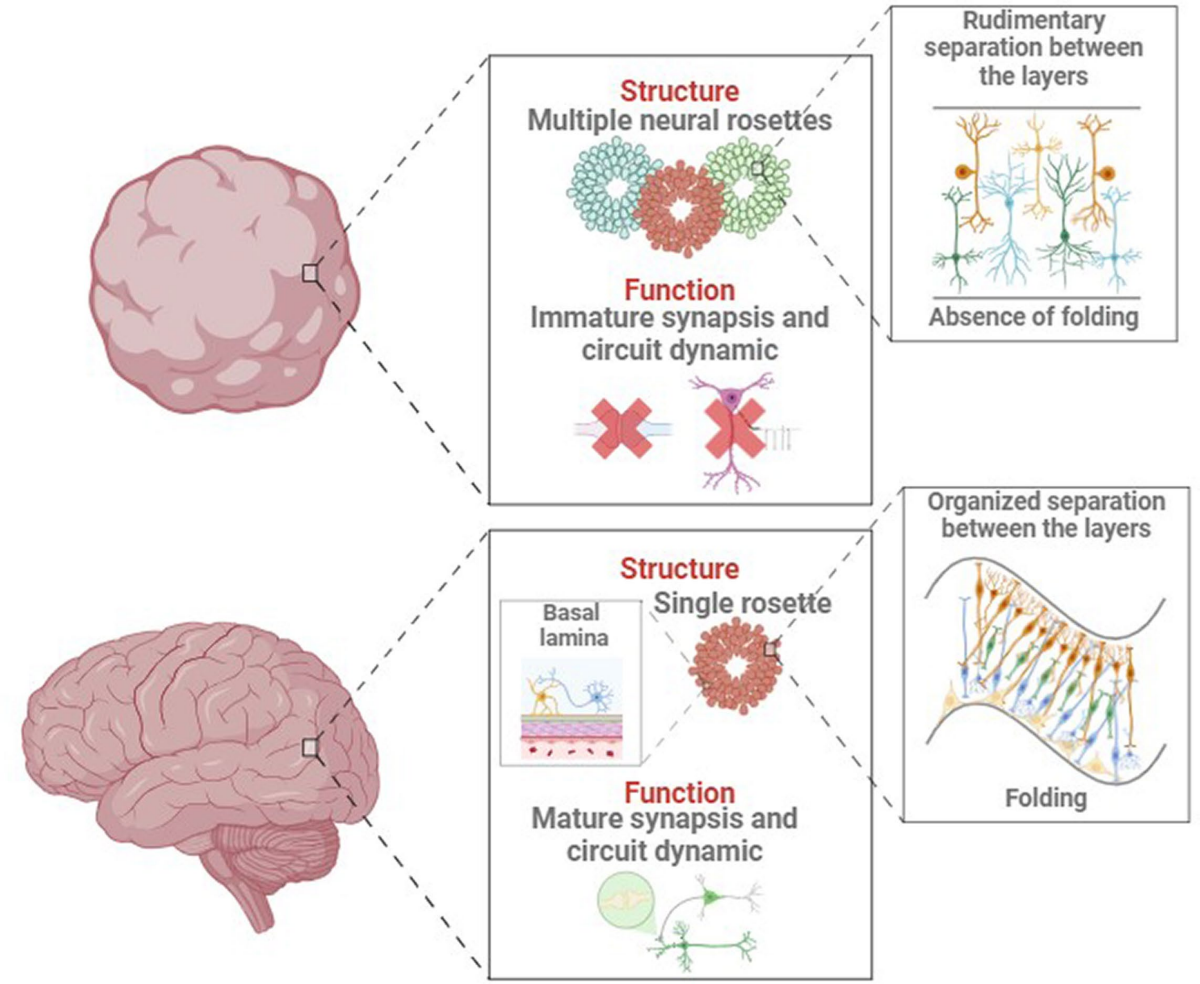
Main structural and functional differences between in vivo and in vitro developing brain and organoid.

Some NDD like AD and PD are characterized by age-related neurodegeneration. Brain organoids naturally recapitulate the early-mid stages of embryonic development (Andrews and Kriegstein, 2022), which precludes reliable modelling of aging and age-associated NDD (Tan et al., 2023). This discrepancy between *in vitro* models and *in vivo* structures might originate from the organoid generation process, as shown in Figure 4. For example, cortical organoids show independent neuroepithelial regions, including the ventricular zone (VZ), subventricular zone (SVZ), and cortical plate (CP), but they lack features of the late-gestational period, such as network formation, myelination, oligodendroglial genesis and microglial population (Quadrato et al., 2016; Tomassy et al., 2016). This must be considered when modeling age-dependent NDDs like AD or PD, where important genetic risk factors and proteins involved in pathogenesis are mainly expressed and produced by glial cells (Zhao et al., 2023). Glial cells and microglia are deeply involved in AD-associated neuroinflammation and amyloid plaque degradation (Fan et al., 2017).

In addition, organoids often show immature dendritic morphology and axonal projection patterns (Zhong et al., 2018). The physiological arborization of midbrain dopaminergic neurons toward the striatum, profoundly implicated in PD pathogenesis, is absent in midbrain models, which lack striatal connections (Sulzer and Surmeier, 2013). Incomplete neuronal maturation compromises functionality, with the absence of prominent sag currents, rebound hyperpolarization, and reduced tonic dopamine release in dopaminergic neurons, as well as (Wilson et al., 1977; Tepper et al., 1995) the formation of pathological aggregates in tauopathies and AD (Medda et al., 2016).

Several strategies, summarized in Table 1, have been proposed to obtain more mature organoids. Simply increasing the culture duration, even if it achieves synapse and circuit maturation, does not result in the activation of the cell senescence program and increases the risk of lower reproducibility (Quadrato et al., 2016). Integrating endothelial and immune cells in organoid cultures is a common strategy to promote aging, but it is technically challenging (Bhaduri et al., 2020). Importantly, both strategies focus on maturation rather than aging, thus they are ineffective for accurately modeling age-dependent NDDs. Nevertheless, pharmacological and genetic approaches have been proposed to accelerate maturation and aging in brain organoids.

**Table 1:** Strategies to improve the maturation and model aging in in brain organoid cultures.

	Benefits	Limitations	References
Prolonged culture periods (more than 250 days)	Mature synapses, dendritic spines and network dynamics. Proper glia differentiation Enable the myelination process	Increases the cost of production and reduces reproducibility	Quadrato et al. (2016)
Co-culture with other cell types	Favours maturation of neurons and circuits formation	Technically challenging, considering different cell types with different proliferation and differentiation rhythms	Bhaduri et al. (2020)
Genetic engineering	Control of proliferation, differentiation and maturation processes in hIPSC	Altered cell physiology inappropriate in translational medicine	Miller et al. (2013), Li et al. (2017), and Ma et al. (2022)
Pharmacological or environmental strategies	Allows induction of aging in hIPSC	Alterations of the natural and physiological cellular mechanisms	Vera et al. (2016) and Kim et al. (2019a,b)

Miller and colleagues engineered human iPSCs from a PD patient to overexpress progerin, a truncated form of laminin A linked with premature senescence (Wheaton et al., 2017). The derived dopaminergic neurons showed genotypic and phenotypic traits typical of the age-related disease (Miller et al., 2013). Using an alternative approach, Vera et al. pharmacologically inhibited telomerase in hiPSCs to increase telomere shortening. Thus, they obtained more senescent dopaminergic neurons with an accentuated PD phenotype; however, telomere length was highly variable among different experiments, even within the same cell line. On the other hand, Li et al. genetically modified human cerebral organoids by enhancing the PTEN-AKT signaling pathway, showing that the deletion of PTEN causes a higher proliferation rate and sensitivity to growth factors in neural progenitors, resulting in expanded size and surface folding in organoids (Li et al., 2017). Lastly, Kim et al. induced oxidative stress and aging by removing antioxidant components from the culture media of human midbrain organoids. After 2 months of differentiation, these constructs showed an increase in the transcription of senescence-associated genes and, in the case of LRRK2 mutant midbrain organoids, PD-associated genes (Kim et al., 2019a). CRISPR-CAS9 technology, already used to introduce mitochondrial DNA mutations in brain organoids (Yang et al., 2019) might be exploited to mimic mitochondrial dysfunctions associated to aging and AD. Unfortunately, all the mentioned strategies may alter the natural differentiation process (Ma et al., 2022).

### 4.2 Functional limitations

#### 4.2.1 Microvascularization

Dual Small Mothers Against Decapentaplegic (SMAD) inhibition, used to generate iPSC-derived organoids, prevents the differentiation of mesodermal cell lineages like endothelial cells and pericytes, thus inhibiting the formation of functional vascularization and limiting the diffusion of nutrients and oxygen to only 200–400 μm deep. The main consequence of the lack of microvascularization and microglia, also of mesodermal origin, is the presence of an inner necrotic core (Pham et al., 2018; Hofer and Lutolf, 2021). Pasca et al. successfully modelled the impact of hypoxia on cortical spheroids (Pașca et al., 2019), observing that hypoxia-related cellular stress affects the number of cortical progenitors reducing organoids viability and size. Moreover, brain organoids showed increased expression of metabolic stress markers like hypoxia inducible factor (HIF-I) (Cakir et al., 2019) and electron transport dysfunction, impairing cellular specification, maturation, morphology and connectivity (Bhaduri et al., 2020; Andrews and Kriegstein, 2022). The neurovascular system is particularly important in neurodegeneration, thus blood–brain-barrier disruption, transcriptional changes in brain endothelial cells, and glymphatic system disruption cannot be studied in non-vascularized brain organoids (Montagne et al., 2017; Rasmussen et al., 2018).

A laborious and skilled experimental paradigm to provide microvasculature to brain organoids is the animal transplantation with host vessels population during organoid differentiation (Mansour et al., 2018). Grafted organoids matured before their *in vitro* counterparts showing late embryonic or early postnatal features (Mansour et al., 2018). Other strategies involving nanotechnology or the use of biomaterials have been exploited to ensure microvascularization and reduce the necrotic core in organoid cultures (Table 2). To study the role of non-neural cells in genetic AD and PD, air-liquid interface after *in vivo* slicing of the organoids, microfluidic devices, and bioreactors have been used to generate IPS-derived brain endothelial cells and mural cells (Berger et al., 2018; Giandomenico et al., 2019). Using these techniques, mutated APOE4 in mural cells has been identified as responsible for amyloid accumulation in the vasculature. While this approach provides important information, the real neuropathogenic role of APOE4 mutation in mural cells must be validated in *in vivo* models (Blanchard et al., 2021).

**Table 2:** Strategies to overcome the “necrotic core” in brain cultures, with their pros and cons.

	Benefits	Limitations	References
Shaking cultures and micro-fluidic devices	Reduced cell death Shaking cultures easy and cheap Enabling continuous medium flow	Do not prevent the formation of the necrotic core	Berger et al. (2018)
Sliced organoids	Recapitulates mature cortical cells subtypes Expression of ALS pathogenic markers in both glia and neurons	Repeated slicing may impair axonal outgrowth 3D partially lost	Szebényi et al. (2021)
Air-liquid interface	Reduced cell death Preserved axonal outgrowth Functional neural circuits present	Late embryonic structures preserved Cortical layers not identifiable 3D partially lost	Giandomenico et al. (2019)
Organoids-on-chip	Functional oxygenation	Expensive method	Salmon et al. (2022)
Direct reprogramming	Improved neuronal functionality Formation of BBB-like structures	Lack of reproducibility	Cakir et al. (2019)
Vascular organoids	Formation of endothelial tubes with smooth muscle cells, pericytes and basement membrane ·Establishment of a network between vessels and neural cells	Lack of BBB specific permeability Lack of a systemic flow	Wimmer et al. (2019)
Hyperoxygenation	Reduced cell death	Physiological condition not represented	Watanabe et al. (2017)
Transplantation in animal models	Reduced cell death Host vascularization	Physiological condition not represented	Popatansov (2018)

Reducing the necrotic core can be achieved by forcing the generation of microvasculature in the organoid through direct reprogramming. Cakir and colleagues performed direct reprogramming of organoid cells into endothelial cells by inducing ETV2 expression, a transcription factor essential for the specification of endothelial and hematopoietic lineages early in gestation (Cakir et al., 2019). The resulting vascularization improved neuronal functionality and cell survival, with minimal apoptosis even at later time points. Astrocytes and pericytes were also found forming, along with the expression of some tight junction markers, a BBB-like structure.

#### 4.2.2 Blood brain barrier

Vascularization of brain organoids can also be induced by generating a blood–brain barrier (BBB) microenvironment using chip technology. A 3D microfluidic platform can be used to vascularize organoids on a chip. This platform consists of a central chamber surrounded by a channel on each side. Brain organoids are placed in the central chamber, while a vascular cell culture containing both endothelial cells and pericytes in the surrounding channels provides the support to generate microvascularization (Salmon et al., 2022). This setup allows interaction between vascular cells and cortical cells. Using this approach, blood vessels can reach the central core, reducing necrosis. Active perfusion assays indicated that the vascular networks were permeable to small compounds, making them useful for testing small membrane-diffusible compounds (Salmon et al., 2022). Microfluidic chips also allow for the control of mechanical stimulation of brain organoids through shear stress, which promotes the penetration of blood vessels into organoids (Cho et al., 2021; Fritschen and Blaeser, 2021), and interstitial flow, which favors the formation of perfusable vascular networks with improved BBB characteristics (Miteva et al., 2010; Winkelman et al., 2022).

#### 4.2.3 Lack of microglia

Microglia, the immune cells of the Central Nervous System (CNS), originate from macrophage progenitors generated in the yolk sac, which migrate and differentiate into the brain around the fourth gestational week (Rezaie et al., 2005; Monier et al., 2007). These cells have a pivotal role in neuroinflammation, a key element in many neurological disorders (Block and Hong, 2005) and most of the commonest NDD (Stephan et al., 2012; O’Rourke et al., 2016; Sekar et al., 2016). Thus, integration of these non-ectodermal cells into brain organoids become imperative to study neuroinflammation-related NDD. Brain organoids with microglia can be obtained using different strategies. First, microglia can spontaneously differentiate into unguided organoids, as these do not use pathway manipulators such as dual SMAD inhibitors. Several groups have demonstrated the presence of cells of mesodermal origin by manipulating culture media composition (Bodnar et al., 2021; Zhang et al., 2023) and Matrigel embedding (Ormel et al., 2018). The microglia-like cells generated showed microglia-specific transcriptomes, phenotypic similarities with *in vivo* microglia, and functional inflammatory and phagocytic responses. However, the main limitation of uncontrolled and spontaneous microglia differentiation is the high variability in the quantity and distribution of different cell types, increasing organoid-to-organoid heterogeneity and reducing replicability (Ormel et al., 2018). Alternatively, endogenous microglia differentiation can be induced in organoids through gene induction systems to overexpress specific transcription factors like PU.1 (Cakir et al., 2022). To increase homogeneity and replicability, other groups introduced exogenous microglia into brain organoids, generating microglia assembloids. In 2017, Abud et al. co-cultured iPSC-derived microglia cells within 12-week brain organoids, which shortly populated the organoids, matured, and responded to neuroinflammatory injury (Abud et al., 2017). Several studies have since replicated this strategy with similar results (Lin et al., 2018; Muffat et al., 2018; Song et al., 2019). However, the self-renewal process manifested by microglia in the brain could not be fully replicated in microglia assembloids (Zhan et al., 2019), limiting the use of this protocol to model chronic NDD. To increase self-renewal cycles in microglia assembloids, several groups have replicated the early migration of macrophage and mesodermal precursor cells from the yolk sac to the CNS (Wörsdörfer et al., 2019; Fagerlund et al., 2022; Sabate-Soler et al., 2022). The main advantage of co-culturing organoids with microglial precursor cells is that the use of mesodermal precursors favors the generation of vascular structures, thereby also addressing the vascularization problem in organoid cultures (Wörsdörfer et al., 2019).

As the timing of integrating microglia and neuroectodermal precursors seems to be determinant (Del Dosso et al., 2020), another interesting alternative is the co-culture of iPSCs or NPCs with macrophagic precursors before generating embryoid bodies. Xu et al. generated regionalized microglia-containing organoids by co-culturing iPSC-derived NPCs and primitive macrophage progenitors (PMPs). This strategy allowed tighter monitoring of the proportions between microglia and other cell types and reduced the appearance of non-neural ectodermal or mesodermal cell types. PMPs used in this protocol developed into functional microglia with phagocytic, inflammatory, and pruning activity observed in the principal NDDs (Xu et al., 2021). Because the state of microglial differentiation and maturity is critical for successful integration into the organoids (Del Dosso et al., 2020), there is no single strategy suitable for all brain organoid protocols. During neurodevelopment, microglia do not migrate uniformly and simultaneously to all brain regions, and these regions do not differentiate at the same time (Menassa and Gomez-Nicola, 2018).

#### 4.2.4 Lack of oligodendrocytes and myelination

In the CNS, myelin is generated by oligodendrocytes, which can form myelin sheath segments for several neurons simultaneously, starting prenatally and continuing through childhood (Poitelon et al., 2020). Mature myelination is a complex event that requires both myelin wrapping and the organization of axonal subdomains, such as paranodal axo-glial junctions and nodes of Ranvier (James et al., 2022). To be functional in modeling demyelinating and most NDD, brain organoid models should generate well-structured and functional myelin sheaths. However, there is currently no consensus on a reliable and functional protocol to model myelination, demyelination, and remyelination. The introduction of Schwann cells in brain organoids would be beneficial for studying ALS, as they are involved in motor neuron myelination and axonal outgrowth processes (Pearse et al., 2007). The lack of myelination in brain organoids may also limit modeling of PD, as the oligodendrocytic population is significantly present in the human midbrain but almost absent in midbrain organoids (Smajić et al., 2022).

Although most regionalized brain organoid protocols result in the innate differentiation of astrocytes and neurons, oligodendrocytes seem to require extra support to differentiate and mature (Marton et al., 2019). To achieve myelinated axons in brain organoids, the protocol should meet the following conditions: generating myelinating oligodendrocytes, generating functionally mature neurons that support myelin ensheathment, and reproducing the embryonic ventral/dorsal signaling—quantity, quality, and specialization.

Recently, Madhavan and colleagues published an innovative two-step protocol specifically aimed at promoting myelination. They used platelet-derived growth factor (PDGF-AA) and insulin-like growth factor (IGF-1) during the early stage of differentiation to promote the expansion of oligodendrocyte precursor cell (OPC) populations, and T3 hormone during maturation to induce the differentiation of OPCs into mature oligodendrocytes and myelination. By week 14, treated organoids showed a robust oligodendrocyte population. By week 30, neuronal axons wrapped with compact myelin were detected, but further structural organization, such as the presence of nodes of Ranvier, was not found. The lack of functional myelination can be attributed to neuronal immaturity and the absence of coherent electrical activity in organoids (Madhavan et al., 2018). In 2021, Shaker and colleagues published a simplified version that resulted in the differentiation of SOX4-positive OPCs in only 4 days while permitting concurrent cortical development, astrocyte differentiation, and neuronal maturation. Treatment with myelination-enhancing molecules, such as clemastine, ketoconazole, and lanosterol, can improve the generation of myelinating organoids, fostering the use of brain organoids to study myelination and remyelination (Shaker et al., 2021). There is increasing evidence that the regional identity of developing oligodendrocytes may influence their mature myelinating properties (Newville et al., 2017). Most protocols attempt to obtain oligodendrocytes in forebrain-regionalized organoids by promoting their differentiation through the “ventral” route, mimicking the first two migratory waves of ventral origin. Current protocols often ignore the leading role that “dorsal” oligodendrogenesis could play in human brain myelination. In 2019, Kim et al. published an innovative protocol based on the independent generation of ventrally and dorsally regionalized forebrain organoids and their subsequent fusion at weeks 5 and 9, respectively. This protocol gave rise to fused forebrain organoids (FFO) that increased the maturation of oligodendrocytes compared to non-FFO (Kim et al., 2019b). Electron microscopy analysis evidenced the presence of compact myelin sheaths, although they showed the same unorganized structure reported previously (Madhavan et al., 2018). Additionally, the expression of genes related to excitatory and inhibitory neurons and synapses was much more balanced in FFOs, promoting the generation of myelinating oligodendrocytes. In Cerneckis and Shi (2023) proposed a modification of the 2D protocol published by Fossati et al. to obtain mature hiPSCs-derived oligodendrocytes (Douvaras and Fossati, 2015). The constructs obtained contained mature myelinating oligodendrocytes and compacted, ultra-structured myelin. Nonetheless, myelination is an adaptive process, highly dependent on neuronal activity (de Faria et al., 2019). Thus, in order to obtain a truly functional model of myelination, we have to consider to promote differentiation and maturation of myelinating oligodendrocytes, as well as the differentiation, maturation and functional communication with the coexisting cell types.

## 5 Engineering limitations

As exemplified in microfluidics for microvascularization strategies, engineering can significantly support the characterization and analysis of brain organoids and assembloids to study neurodevelopmental disorders (NDD). In this context, engineering investigations are crucial for quantitatively: (i) comparing cerebral organoids to human brain samples to assess their similarity to their *in vivo* counterparts, and (ii) describing any alterations between *in vitro* constructs derived from cells of healthy individuals and those with Parkinson’s Disease (PD). Moreover, these quantitative evaluations are fundamental prerequisites for their robust mass production (Choudhury et al., 2020), and necessary for the creation of an international organoid biobank (Brémond Martin et al., 2021). Most limitations, particularly those related to structural and functional analysis, have been identified in a previous review (Poli et al., 2019).

The literature presents various methods, tools, and algorithms, some specifically developed for processing datasets representing human brain organoids (e.g., Brémond-Martin et al., 2023; Deininger et al., 2023). However, to the best of our knowledge, there is a lack of quantitative descriptions of cell morphology and arrangement, as well as network dynamics of brain organoids, and a comparative analysis between healthy and diseased constructs. Instead, there are rather qualitative or raw considerations about differences between the two groups (e.g., Smits et al., 2019).

## 6 Ethical issues

The promises and potential of organoids and assembloids in providing insights into NDDs inevitably come with ethical discussions regarding their social benefits in the short and long term. Beyond the classic ethical issues of cell donor consent, long-term storage, utilization, and the feedback of clinically relevant information to the patient, which must be strictly regulated by ethical and legal requirements (Bredenoord et al., 2017), further regulations are necessary. Above all, brain organoid technology has sparked significant debate about monitoring, which might reveal intimate information about the donor, ranging from early-stage indicators of neurodegeneration to consciousness and sentience. Although current organoid technology cannot exhibit higher-level brain functions (Lavazza and Massimini, 2018), they contain mature neuronal populations, establish functional and active neuronal networks, and can respond to optical sensory stimulation; thus, they may be able to integrate information.

## 7 Conclusion

### 7.1 *Pros* and *cons* of using human brain organoids to model NDDs

Human brain organoids have significant potential in modeling the onset and progression of NDDs. Their use in neurodevelopmental biology and associated pathologies, as well as to study rare genetic diseases, has become clear. However, modeling age-dependent NDDs still presents evident functional and technical limitations. Considering their use as an alternative to animal models is a radical assumption that is still far from reality. Animals still represent the most complex systems for studying specific fields like behavior, neurological diseases, or psychiatric disorders. However, their use in biomedical research is set to be drastically reduced due to ethical issues, strict laws and guidelines, and societal pressure. On the other hand, brain organoid research is growing rapidly in terms of publications and funded projects, making their use in neuroscience a promising and necessary complement to understanding the molecular and cellular basis of neurodevelopment and brain pathology. Looking forward, we must consider the following pros and cons to establish a solid mainstream in neuroscience research.

*Main pros*: (1) These miniature models offer a unique opportunity to replicate human brain structure, maintaining the genetic fingerprint of donor cells. (2) Organoids can be analyzed using interdisciplinary approaches from molecular biology, immunological assays, electrophysiology, and imaging. (3) A single preparation can produce hundreds of organoids, which can be used for drug screening to identify novel therapeutic targets in a controlled and ethical manner. *Main cons*: (1) Overmaturation of brain organoids never achieves the activation of the aging program but increases cell death and heterogeneity. (2) Functional studies can be affected by the poor representation of the complete CNS cell set. Effective protocols to generate brain organoids with the exact cell composition of ectodermal (e.g., myelinating oligodendrocytes) and mesodermal origin (e.g., microglia, endothelial cells) are still poorly developed. Consequently, brain organoids lack microvascularization, clearance of dead cells, and have extensive necrotic cores.

Therefore, the use of brain organoids to model age-dependent NDDs needs improvement. The combination of protocols that consider exact developmental waves and the incorporation of functional cells of mesodermal origin will bring a simple 3D model closer to the human brain. The presence of microglia and a functional microvasculature is not only important to reproduce a reliable brain model but also to mimic the functioning of the human brain during development, supporting neurogenesis and the proliferation of neural progenitors, and maintaining the balance between synapse formation and pruning. For example, microglia are also indispensable in the adult brain, where they support neuronal maturation, modulate neural activity, and contribute to maintaining correct homeostasis. In recent years, biotechnology has helped to find new strategies to overcome these limitations. Implemented solutions like microfluidics, organoid-on-chip, or the development of new matrix substrates, together with improved protocols aimed at replicating neurodevelopment, will pave the way to generate aged brain organoids.

## Author contributions

NU-A: Investigation, Writing – original draft. SC: Investigation, Writing – original draft. FC: Conceptualization, Supervision, Writing – original draft, Writing – review & editing. CM: Conceptualization, Supervision, Writing – original draft, Writing – review & editing.
